# A randomised study of nurse collected venous blood and self-collected dried blood spots for the assessment of cardiovascular risk factors in the *Understanding Society* Innovation Panel

**DOI:** 10.1038/s41598-023-39674-6

**Published:** 2023-08-10

**Authors:** Meena Kumari, Alexandria Andrayas, Tarek Al Baghal, Jonathan Burton, Thomas F. Crossley, Kerry S. Jones, Damon A. Parkington, Albert Koulman, Michaela Benzeval

**Affiliations:** 1 Institute for Social and Economic Research, University of Essex, Colchester, UK; 2 School of Life Sciences, University of Essex, Colchester, UK; 3 School of Psychological Science, University of Bristol, Bristol, UK; 4 European University Institute, Fiesole, Italy; 5 Nutritional Biomarker Laboratory, MRC Epidemiology Unit, University of Cambridge, Cambridge, UK

**Keywords:** Cardiovascular biology, Risk factors

## Abstract

Dried blood spot (DBS) sample collection has been suggested as a less invasive, cheaper and more convenient alternative to venepuncture, which requires trained personnel, making it a potentially viable approach for self-collection of blood on a large scale. We examine whether participants in a longitudinal survey were willing to provide a DBS sample in different interview settings, and how resulting cardiovascular risk biomarkers compared with those from venous blood to calculate clinical risk. Participants of the *Understanding Society* Innovation Panel, a representative sample of UK households, were randomly assigned to three modes of interview. Most participants (84%) were interviewed in their allocated mode. Participants (n = 2162) were interviewed by a nurse who collected both a blood sample by venepuncture and a DBS card (‘nurse collection’) or participants were seen by an interviewer or took part in the survey online to self-collect a DBS card (‘self-collection’). All DBS cards were returned in the post after the sample had dried. Lipids (total cholesterol, HDL-cholesterol, triglycerides), HbA1c and C-reactive protein were measured in venous and DBS samples and equivalence was calculated. The resultant values were used to confirm equivalent prevalence of risk of cardiovascular disease in each type of blood sample by mode of participation. Of participants interviewed by a nurse 69% consented to venous blood sample and 74% to a DBS sample, while in the self-collection modes, 35% consented to DBS collection. Demographic characteristics of participants in self-collection mode was not different to those in nurse collection mode. The percentage of participants with clinically raised biomarkers did not significantly differ between type of blood collection (for example, 62% had high cholesterol (> 5 mmol/l) measured by venepuncture and 67% had high cholesterol within the self-collected DBS sample (*p* = 0.13)). While self-collected DBS sampling had a lower response rate to DBS collected by a nurse, participation did not vary by key demographic characteristics. This study demonstrates that DBS collection is a feasible method of sample collection that can provide acceptable measures of clinically relevant biomarkers, enabling the calculation of population levels of cardiovascular disease risk.

## Introduction

Cardiovascular disease is the leading cause of death worldwide. In the UK, cardiovascular disease is patterned by several social and environment factors^1,2^. The inclusion of a cost-effective, low-resource demanding blood collection method in population-based studies would advance research to understand how social and environmental factors affect biological pathways that may mediate these associations. Blood specimens from non-clinical population-based social surveys are often lacking due to the financial and practical demands on the survey and participants of traditional blood collection through venepuncture by skilled health care professionals.

Standard protocols for blood sample collection require face-to-face contact. Most commonly, collection of bio-specimens for research takes place in a clinic and/or the participant’s home by a trained phlebotomist or nurse. Research clinics, typically a medical or research facility or ‘mobile clinic’, provide expert medical personnel, phlebotomists, and specialised equipment to ensure the appropriate handling of samples^3–5^. This method of blood sample collection is considered optimal as it allows for standardised and immediate processing of samples and provides opportunity for additional tests. However, this method of data and sample collection is labour intensive, expensive compared to other methods and participants may be unable or prefer not to travel, biasing the characteristics of the sample^6^. Alternatively, participants may be visited in their own homes by trained nurses. This method has the advantage of reducing bias^6^ and potentially reducing costs compared with clinic-based studies^7^ but complicates immediate standardised processing of samples.

The resource challenges of the above approaches can be addressed by dried blood spot (DBS) sampling, which is a low resource-demanding alternative to venous blood draws, and as less invasive, may be more acceptable to participants. DBS samples are small volumes of capillary blood, obtained by fingerprick and blotting of the blood onto filter paper. This method, which has been used in new born screening programmes, reduces personnel and shipping costs, and is an accepted means of collecting blood samples in a research setting^8^.

A number of social studies such as National Social Life, Health, and Aging project^8^, Health and Retirement study^9^ and INTERHEART^10^ have adopted this approach. These studies have measured a number of cardiovascular risk factors, including total cholesterol, high density lipoprotein (HDL) cholesterol, glycated haemoglobin (HbA1c) and C-reactive protein (CRP). A systematic review identified 16 studies that compared DBS with venous measurement of HbA1c and lipids^11^ and suggested a number of factors related to storage that influence the comparability of results between DBS and venous blood samples. Crimmins et al.^12^ reported that total cholesterol and HDL cholesterol measurements were associated with the time between sample preparation and measurement. This is important in studies that may wish to ascertain clinical burden within a population. However, other studies have suggested the time between sample collection, processing and measurement may be less important for HbA1c^13^ and CRP^14^.

For the successful use of DBS in population surveys, it needs to be demonstrated that the characteristics of participants that complete a DBS collection are not systematically different compared to those that provide venous blood. Further, to enable population level estimates of disease, it is necessary to determine whether analyte concentrations measured in DBS and venous blood samples are equivalent. To address these issues, we included DBS and venous blood sample collection in a randomised study of the general population. The aims of this study were (a) to describe the demographic characteristics of participants that took part in the survey, consented to give venous and/or DBS samples, and who provided good quality DBS samples; (b) to estimate serum-equivalent DBS values and compare results to venous samples, investigating how blood sample characteristics affects the agreement between the two measures; c) to investigate if serum-equivalent DBS measures are suitable to assess clinical risk.

## Methods

This paper uses data collected in the *Understanding Society* Innovation Panel (IP), which is a stratified clustered random sample of households, representative of Great Britain with the exclusion of the northernmost part of Scotland. It is used to test methodological innovation in longitudinal surveys with annual data collection from everyone aged over 10 years^15^. The IP was initially composed of 1500 households with subsequent refreshment samples in waves 4, and 7 and 11. Data are used from the twelfth wave of the Innovation Panel 12 (IP12), collected in 2019. All methods were carried out in accordance with Information Commissioner’s Office guidelines and regulations. The study was reviewed and approved by the NHS Health Research Authority: East of England—Essex Research Ethics Committee, REC reference: 19/EE/0146**.** Informed consent was obtained from all participants and/or their legal guardian(s)**.**

### Sample processing

**Figure 1:**
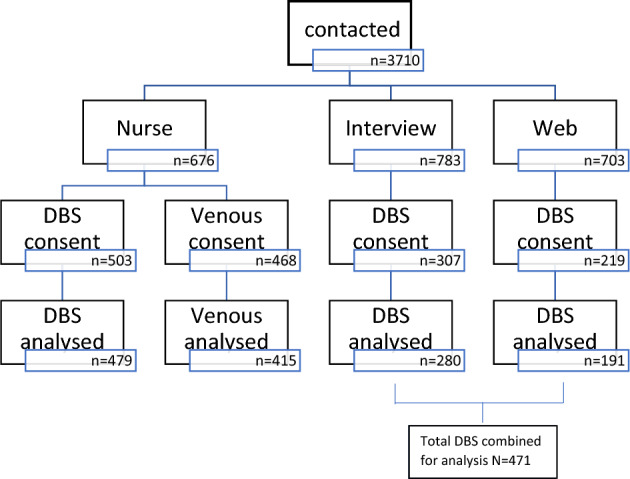
Flowchart showing IP12 recruitment and consent to provide blood samples.

In IP12^16^, households were randomly assigned to three modes of interview; a face-to-face nurse interview, a face-to-face interviewer interview, or web administered data collection. Modes and interviewers were arranged over the phone and participants who refused a face-to-face interview were offered a web administered interview and those who did not take up the web survey were offered a face-to-face interview by a social interviewer*.* For participants interviewed by a nurse, a 6 ml serum tube and a 4 ml EDTA whole blood tube was collected from those aged 16 + years and posted at ambient temperature to the MRC Epidemiology Unit Biorepository, Cambridge for processing, aliquoting, storage and analysis. DBS samples were also collected. It was established in an earlier study that it would take longer than a typical interview to dry the blood spot^17^ and thus samples and a return envelope were left with the participants to post to the University of Essex. With interviewer administered data collection, if the participant agreed to take a DBS sample, instructions and return envelopes were provided and a sample kit left with them to return to the University of Essex. Participants in the web administered data collection mode were asked during the survey if they wanted a sample collection kit to be sent to them. The collection and link to instructions are available here (https://www.understandingsociety.ac.uk/blood). Participant numbers for recruitment and consent to provide blood samples are shown in Fig. 1. For the purposes of analyses presented in this paper, DBS samples collected by participants (whether requested during a face-to-face interview or via the web survey) were considered self-collected samples.

After reception at MRC Epidemiology Unit, venous blood samples for glycated haemoglobin were refrigerated until analysis. The remaining venous blood samples were centrifuged, and aliquots of plasma and serum were stored at − 70 °C until analysis. DBS samples were received at the University of Essex, stored at room temperature, batched and sent to MRC Epidemiology Unit where they were later frozen at − 70 °C until analysis. DBS samples were stored in small plastic bags with a sachet of desiccant. The DBS samples were graded by visual inspection and given two scores: (1) number of useable spots (0–5 useable spots or missing if no spots) and (2) if applicable, a quality code to describe the spots (operationalised as either: good quality with no issues, not dried, other issue, or not dried and additional issue). The latter is based on commonly used DBS grading charts^18^. The term ‘useable’ refers to any sample that was sufficient for measurement of any analyte.

### Analytes

Total cholesterol, triglycerides and CRP from serum and DBS and glycated haemoglobin from whole blood or serum and DBS were measured using a Siemens Dimension Xpand clinical chemistry analyser (Siemens Healthcare Ltd, Camberley, Surrey, UK). Method details including extraction from DBS, as well as reagents, control quality materials and QC information can be found in Supplementary Materials 1–2. Throughout, the term ‘venous’ blood sample may refer to either the serum or whole blood result, depending on the analyte.

Except for CRP, the use of third-party reagents (Randox Laboratories, County Antrim, UK) allowed assays to be programmed manually on the analyser and enabled the parameters recommended for serum assays to be adapted for the higher sensitivity required for DBS analysis. This approach kept the serum and DBS assays as closely aligned as possible to reduce bias between methods. CRP was assayed using the Siemens Dimension Xpand CardioPhase® high sensitivity assay. Serum CRP was measured according to the manufacturer’s instructions. To use the same CRP method for DBS, the assay was calibrated using a 50-fold dilution in PBS of the manufacturer’s recommended kit calibrator (CCRP calibrator). This dilution was chosen to be equivalent to the CRP in the DBS eluent. In all other aspects the assay was run using the manufacturer’s protocol for serum CRP and provided equivalent results for the sample types (Supplementary Materials 2).

Measurements of triglycerides, HbA1c and CRP did not meet the assumption of normality assessed using Shapiro–Wilk tests. To overcome this, triglyceride and CRP data were log transformed and inverse measures for HbA1c were used for equivalency. (Supplementary Materials 3). Venous and DBS based measures that were 3 standard deviations above or below the mean per analyte were excluded from analysis.

### Covariates

For both venous and DBS results, the time a sample was kept at room temperature was calculated as the time between sample collection by the nurse/participant and receipt at MRC Epidemiology Unit. All venous blood samples were processed before COVID-19 related laboratory closure. A variable was included to describe whether DBS samples were measured before or after the COVID-19-related lab closure. Other indicators of the quality of the DBS sample considered were the visual inspection score described above and the number of blood spots on the DBS card.

Responses to questionnaires were used to record age, sex, highest level of education (obtaining a degree or higher *vs.* not), self-rated health (excellent/good vs. poor) and long-standing illness (yes vs. no), meaning anything that has troubled the participant over a period of at least 12 months or is likely to over a period of at least 12 months. Participants were also asked if a doctor had ever diagnosed them with diabetes (yes vs. no).

### Statistics

Descriptive statistics were used to compare response rates to the overall survey, consent rates for providing each type of blood sample and the provision of ‘good’ quality DBS samples by mode of interview. The population characteristics of participants who gave consent to give a venous blood sample, consent to a DBS blood sample, and provided a good DBS sample, by actual mode of interview, were compared using the Wilcoxon rank sum test for continuous variables and Pearson's Chi-squared test for categorical variables.

To calculate venous equivalent-DBS values, for those participants with both samples, venous results were regressed on DBS raw sample values using Deming regression. This was carried out using the mcr R package^19^. Deming regression^20,21^ finds the line of best fit by minimizing the sum of the distances between the measured values and the regression line at an angle specified by the variance assuming both variables are measured with error. In this case the default variance ratio of 1 was used which defines the residual as the perpendicular distance from a point to its fitted value and this is deemed acceptable when the range of measurements is large compared to the measurement error as is the case here. The Deming model parameter estimates for slope and intercept were used to calculate a venous-equivalent value for each DBS value. Inverse measures of HbA1c were converted back to their original units while geometric means and standard deviations are reported for the log-transformed triglycerides and CRP data.

To investigate how closely the raw DBS measures match those taken from venous blood or serum, where both measures were taken from the same participant, simple linear regression was used to compare venous or serum values with—venous-equivalent DBS values (Supplementary Materials 4). This was used to estimate the variance in venous concentrations explained by DBS values for total cholesterol, CRP, triglycerides and HbA1c and to obtain R-square values. In addition, Pearson bivariate correlations were estimated to assess the strength of the relationship between DBS venous-equivalent and venous values, and root mean square errors were calculated to estimate the accuracy of DBS venous-equivalent values. Averages of each analyte by mode were also calculated (Table 3).

Bland–Altman plots (Supplementary Material 5) were used to visualise and assess the agreement between the two blood sample types whereby the average analyte measures between venous-equivalent DBS and venous values are plotted against difference scores between the two measures^22^. A positive (or negative) mean difference represents a venous-equivalent DBS overestimation (or underestimation) of the venous based biomarker and the degree of scatter in difference values on the Y-axis indicates the amount of variability between sample types.

DBS and venous sample characteristics were investigated to understand if these impacted the agreement of raw DBS measures to venous-equivalence; analysis of variance was used to measure differences between a base model that included only the DBS values regressed on venous values, against another model where for each indicator of blood sample quality an interaction term between DBS and the covariate in question was included (Supplementary Material 6).

Cut-offs were used to estimate CVD risk in the venous-equivalent DBS measures; for HbA1c diabetes risk was defined as greater than 6.5%, high cholesterol at a threshold of 5 mmol/l, and high CRP at 10 mg/l. Pearson's Chi-squared tests were used to compare the proportion of participants’ with diagnosed diabetes and participants’ analyte levels that were above clinical cut-offs between the self-collected DBS samples and venous bloods collected by nurses. These analyses were carried out unweighted and weighted to adjust for the complex sample design and likelihood of being included in the Innovation Panel^23^.

### Ethics approval

The University of Essex Ethics Committee has approved all data collection on *Understanding Society* main study and innovation panel waves. ‘Understanding Society Health Innovation Panel: Biomeasure and health data collection from the Innovation Panel of the UK Household Longitudinal Study’ specifically covering the collection of the data used in this manuscript was approved by East of England—Essex Research Ethics Committee, Ref 19/EE/0146, 18th June 2019.

## Results

Following contact, a number of participants failed to participate in the study. Reasons for failure to participate are shown in Supplementary Material 7. Thirty three participants contacted to take part in nurse mode requested to take part in self-collection mode. While there was movement between the self-collection modes, all participants allocated to a self-collection mode remained in this mode.

### Characteristics of participants who gave a full interview, consented to venous or DBS samples and provided a good DBS sample by mode of interview

In actual or realised mode, consent rates for biological sample collection were higher in the nurse mode (74%) than in those invited for self-collection (35%). Participants reported they were likely to take part again (likelihood of participating again score from 1 to 10: 8.8 ± 1.7) irrespective of whether they were seen by a nurse (8.8 ± 1.8) or not (8.9 ± 1.6).

With the exception of age, there were no differences in the characteristics of those who consented to a DBS sample by nurse or self-collection (Table 1). There were no significant differences in the characteristics of participants who gave a ‘good’ quality DBS sample between nurse or self-collected mode. Within self-collection, the proportion of men collecting a good sample was lower than those completing the initial questionnaire (*p* < 0.05). There was a difference between the nurse and self-collection modes in the proportion of participants with a long-standing illness who gave a full interview (*p* = 0.006), with fewer participants stating long-standing illness in the self-collection mode compared to those seen by a nurse, but this was not apparent in those who consented to give a DBS sample or gave a good quality DBS sample.

**Table 1:** Participant characteristics of those who gave a full interview, consented to blood sample, and provided a good DBS sample by mode of interview/DBS sample collection.

Characteristic	Nurse					Self-collection		
	Full interview	DBS consent	Good quality DBS	Venous consent	DBS and venous analysed	Full interview	DBS consent	Good quality DBS
N	676	503	382	468	384	1486	527	222
Age, mean (SD)	53.3 (18.0)	53.0 (17.5)	54.0 (16.9)	53.6 (17.0)	54.3 (16.7)	52.3 (18.3)	56.4 (16.7)***	54.6 (17.4)
Male, n (%)	306 (45%)	221 (44%)	168 (44%)	206 (44%)	178 (46%)	672 (45%)	229 (43%)	84 (38%)*
Degree or higher, n (%)	283 (43%)	230 (46%)	173 (46%)	206 (44%)	168 (44%)	624 (43%)	239 (46%)	105 (48%)
Good self-rated health, n (%)	479 (74%)	367 (76%)	281 (76%)	340 (75%)	279 (75%)	1080 (74%)	380 (72%)	163 (74%)
Long standing illness, n (%)	278 (41%)	200 (40%)	152 (40%)	193 (41%)	165 (43%)	521 (35%)**	201 (38%)	88 (40%)
Doctor diagnosed diabetes, n (%)	62 (9.8%)	47 (9.8%)	37 (10%)	43 (9.7%)	31 (8.5%)	122 (8.6%)	55 (11%)	24 (11%)

*p < 0.05, **p < 0.01, ***p < 0.005 vs equivalent consent in nurse mode. ^a^*p* < 0.05 vs full interview in self collection.

### Characteristics of DBS and venous samples by mode of sample collection

A higher proportion of nurse collected DBS samples were rated good quality compared with self-collected DBS, 80% vs. 47% (*p* < 0.001), respectively. Self-collected samples were kept at room temperature for a shorter time (*p* < 0.001) than those collected in the nurse mode, which was conducted earlier in the study (Table 2).

**Table 2:** DBS and venous characteristics by final mode of interview limited to those whose DBS samples was analysed.

Characteristic	Nurse	Self-collection	Overall
N	479	471	950
Number of spots per card, mean (SD)	3.59 (1.40)	3.40 (1.50)	3.50 (1.45)
Quality of spots, n (%)			
Good	382 (80%)	222 (47%)	604 (64%)
Not dried	69 (14%)	134 (28%)	203 (21%)
Other issue	25 (5.2%)	88 (19%)	113 (12%)
Not dried and additional issue	3 (0.6%)	27 (5.7%)	30 (3.2%)
Laboratory batch*, n (%)			
Before closure	342 (71%)	259 (55%)	601 (63%)
Delayed processing	137 (29%)	212 (45%)	349 (37%)
Days DBS at room temperature, mean (SD)	73 (23)	51 (15)	62 (22)
Days between venous collection and lab delivery, mean (SD)	3.47 (1.73)	–	3.47 (1.73)

### DBS to venous measurement and equivalency

In the venous and DBS samples where both samples yielded a measurement, for total cholesterol, CRP, triglycerides and HbA1c, there was a significant relationship between venous and DBS measures (*p* < 0.0001). The association was not present for HDL cholesterol (Adjusted R^2^ = 0.005, *p* = 0.08) (Supplementary Materials 8) so this analyte was not included in further analyses.

For total cholesterol, CRP, triglycerides and HbA1c the following DBS-to-venous (serum or whole blood) equivalency equations were obtained by regressing serum or whole blood values on raw DBS values using Deming regression.

$$Y_{{Total{ }cholesterol}} = 0.95\left( {DBS} \right) - 0.15$$

$$Y_{CRP} = 0.44\left( {DBS} \right) + 1.66$$

$$Y_{Triglycerides} = 1.77\left( {DBS} \right) - 2.43$$

$${ }Y_{HbA1c} = 1.14\left( {DBS} \right) + 0.02$$

The mean and standard deviation for venous and venous-equivalent DBS values for each analyte, by mode of sample collection, are shown in Table 3. Scatterplots (Supplementary Materials 4) show venous regressed onto venous-equivalent DBS measurements for total cholesterol, CRP, triglycerides and HbA1c. The bivariate correlations between venous and serum-equivalent DBS values were 0.49 for total cholesterol, and 0.87 for CRP, 0.37 for triglycerides, 0.41 for HbA1c.

**Table 3:** Total cholesterol, C-reactive protein, triglycerides, and glycated haemoglobin values for venous and serum-equivalent DBS data.

Analyte	Nurse (Venous)		Nurse (DBS)		Self-collection (DBS)	
	N	Mean (SD)	N	Mean (SD)	N	Mean (SD)
Total cholesterol (mmol/l)	414	5.36 (1.06)	464	5.32 (1.05)	449	5.49 (1.04)
Haemoglobin A1c (%)	388	5.61 (0.68)	470	5.53 (0.76)	455	5.51 (0.76)

Analyte	Nurse (Venous)		Nurse (DBS)		Self-collection (DBS)	
	N	Geometric mean (GSD)	N	Geometric mean (GSD)	N	Geometric mean (GSD)
C-reactive protein (mg/l)	396	1.88 (2.84)	278	3.81 (1.88)	277	3.73 (1.83)
Triglycerides (mmol/l)	408	1.44 (1.54)	460	1.43 (1.82)	448	1.14 (1.98)

### Agreement between venous and DBS measures

Bland–Altman plots were used to assess agreement between venous-equivalent DBS compared to venous measures (Supplementary Materials 5). The median error observed between venous-equivalent DBS and venous values measured 0.03 mmol/l, 0.15 mg/l, 0.05 mmol/l and 0.19% for total cholesterol, CRP, triglycerides, and HbA1c respectively. The average bias measured 0.02 mmol/l (± 1.03), 0.25 mg/l (± 1.38), − 0.15 mmol/l (± 1.07) and 0.15% (± 0.73). No significant systematic bias in total cholesterol (R^2^ = − 1.70e−03, F(1, 370) = 0.37, *p* = 0.543) and HbA1c (R^2^ = − 2.78e−03, F(1, 353) = 0.02, *p* = 0.893) between venous-equivalent and venous measures was observed. However, a statistically significant proportional bias was observed in CRP (R^2^ = 0.21, F(1, 206) = 57.04, *p* < 0.001) and triglycerides (R^2^ = 0.18, F(1, 365) = 81.83, *p* < 0.001). These observations suggest venous-equivalent DBS measurements of total cholesterol and HbA1c are reliable however a proportional bias exists in measures of CRP and triglycerides where values of triglycerides become increasingly over-estimated and CRP become increasingly under-estimated with increasing concentration.

### DBS and venous characteristics associated with agreement between measurements

Of the DBS characteristics covariates tested, all impacted the association between serum and DBS measurements of at least one analyte. This means that models with an interaction between DBS values and the covariate in question significantly differed to the same nested model without said interaction. The number of spots influenced the agreement between venous and venous-equivalent DBS measures of HbA1c, the quality of DBS spots influenced the agreement between CRP measures, whether sample processing was delayed impacted HbA1c and total cholesterol, the number of days between collection of the DBS sample and − 70 °C storage also impacted HbA1c and total cholesterol such that adverse conditions tended to be associated with higher equivalence values. Days between venous blood collection and lab delivery was not significantly associated with any analyte (Supplementary Material 6). Outputs of the full linear models with interaction terms for each covariate can be found in Supplementary Material 6.

### Using DBS data to assess disease prevalence

Using total cholesterol, HbA1c and CRP data, we compared the prevalence of disease in venous and venous-equivalent DBS. Comparable measurements should lead to a similar prevalence of disease in nurse and self-collection mode. However, participants who reported doctor diagnosed diabetes were less likely to provide a venous sample than take part in the nurse home visit. In the population that provided a venous sample, reported any diabetes diagnosis and had HbA1c values measured, 5.7% of participants report that a doctor told them they have diabetes compared to 8.7% who had diagnosed diabetes in those that provided a self-collected DBS sample and had their HbA1c values analysed (Table 4). 7.6% of participants who gave a venous sample and 12% of participants who gave a self-collected DBS sample had HbA1c levels above the 6.5% threshold. Applying survey weights failed to substantially alter any conclusions. Also, in survey-weighted proportions, used to enable population level inference, there is a significant difference in the proportion of participants above the 6.5% clinical cut-off for HbA1c.

**Table 4:** Unweighted and weighted percentages for those diagnosed with diabetes and above clinical cut points in serum-equivalent DBS based measures of HbA1c, total cholesterol and C-reactive protein.

Characteristic	N	Unweighted				Weighted			
		Nurse (Venous)	Self-collection (DBS)	X^2^	*P*	Nurse (Venous)	Self-collection (DBS)	X^2^	*P*
Doctor diagnosed diabetes	807	21 (5.7%)	38 (8.7%)	2.57	0.11	23 (6.3%)	37 (9.3%)	2.32	0.13
Above 6.5% HbA1c	807	28 (7.6%)	51 (12%)	3.64	0.06	27 (7.5%)	52 (13%)	5.76	0.02
Above 5 mmol/l cholesterol	863	257 (62%)	301 (67%)	2.32	0.13	233 (57%)	269 (63%)	3.27	0.07
Above 10 mg/l CRP	673	14 (3.5%)	8 (2.9%)	0.22	0.64	10 (2.6%)	7 (3.1%)	0.10	0.75

In venous-equivalent DBS measurements an overestimation of disease for diabetes prevalence may have occurred. However, there may also be an underestimation of diabetes in the nurse visit as the prevalence of doctor diagnosed diabetes is lower than in DBS arm of the experiment. Further, it was noted that higher proportion of HbA1c values above 3SD were found in those reporting doctor diagnosed diabetes in the nurse visit than in from those reporting doctor diagnosed and providing a DBS (Supplementary Materials 9). The proportion of participants above clinical thresholds for the additional cardiovascular risk factors did not significantly differ by mode of interview in unweighted or weighted analyses, which account for movement between allocated and actual mode of participation.

## Discussion

Our findings suggest that, while response rates for self-collected DBS samples are around half that in a nurse visit, characteristics of participants are unbiased with respect to educational attainment, self-rated health and longstanding illness. While measurement quality of HbA1c and HDL-cholesterol may be inconsistent by DBS, a high proportion of DBS samples were classified as ‘good’, and DBS can be a cost-effective alternative in large-scale surveys/studies to assess sub-clinical and clinical disease burden.

Our findings suggest that DBS collection is a feasible collection method. The analyses presented generally accord with findings from other population studies that have introduced self-collection and observed reduced response rates^24^. There was no participant selection bias based on the characteristics of those that consent by age, educational attainment which accords with earlier studies^25,26^. It has been reported that chronic conditions such as diabetes are associated with lower levels of consent and this might have been expected, given the analytes we measured in the study. This is apparent for the Health and Retirement study (HRS)^25^ and the SHARE study^26^ who describe odds ratios of 1.27 for failure to consent in those with diabetes. Our findings accord with this, but it appears to be specific to diabetes. Our findings show a difference in long-standing illness between those that took part in a full interview in the nurse visit compared to self-administered collection however differences in health status were not observed within the population who consented to give a DBS sample. This suggests participants with a long-standing illness in the self-collection mode may be less likely to take part in the survey initially compared to the nurse mode, but they were not less likely to give a blood sample. Our observations may relate to methodological differences as we randomised participants to receive a nurse visit or self-collection and could account for change in actual mode while other studies described the consent rates following introduction of the collection. It may also relate to the different age profile of HRS and SHARE, which are studies of older people for whom health might be more salient than for younger people^27^ as we saw an association of consent rates with age. Others have demonstrated a loss of response from initial invitation, to participation and to blood collection by nurses in the home^28,29^, and that this final loss of response can vary by health status^30^.

We developed reliable methods for the collection of DBS and measurement of total cholesterol, triglycerides, CRP and HbA1c in DBS but less successfully for the measurement of HDL-cholesterol in accordance with some^14,17,31–33^ but not all^34^ previous research. This may be related to stability of HDL cholesterol (and to a lesser extent cholesterol) in the DBS samples. We considered DBS quality checks, which were associated with measures as previously described^12^. We had previously attempted to collect DBS in a study where nurses remained with participants during sample drying but this was unsuccessful as a percentage of spots were not dried^17^, which impacted measurements^12^. In the current study, participants were left the completed DBS card or were asked to collect their own sample. This approach proved successful resulting in a large proportion of samples that were considered of good quality. However, our data suggest that poor quality samples may have introduced noise as analyte values varied by the quality of DBS sample collected. Further improvement could be obtained through better training of web respondents, where a reduced proportion of good quality samples were obtained. Further, due to cost constraints, we could not standardise conditions, such as temperature or humidity at home or during transport, previously described as impacting DBS quality^35^.

Despite these constraints, equivalence of the DBS data to predict prevalence of disease from the self-collected study samples suggested that equivalence was sufficient for us to accurately describe population prevalence of hypercholesterolaemia and elevated CRP^33,36^. The use of HbA1c may overestimate diabetes disease prevalence, however in our data HbA1c-dereived estimates were not significantly different from prevalence of doctor diagnosed.

Our study has a number of strengths. Unlike previous studies we randomly assigned households to receive a nurse visit to collect a venous and DBS sample. Our findings of similar clinical characteristics by mode of collection, as would be expected in a well randomised study, provide more robust findings of equivalence. Thus, our study is not subject to the biases associated with earlier observational studies. Our study is large, spans the adult age range and our sampling strategy means that we can make population level inferences through the use of inverse probability weighting. The analysis of a proportion of samples was delayed with the closure of laboratories during the COVID-19 pandemic, which enabled us to examine delay of measurement in our analyses previously described to impact integrity of measurements in the DBS^37^. Our analytical method for DBS samples was not successful for the measurement of HDL cholesterol and this requires further refinement but was at least in part related to stability of HDL cholesterol on the DBS. Our data indicate that further work is needed on participant instructions to improve self-collection where there was a lower proportion of useable samples that were deemed ‘good’ than when samples were collected by trained nurses. The equivalency equations are specific to this study and may not be applicable to other studies due to a lack of standardisation of protocols. Future work to harmonise these equations may serve to provide equivalence equations to enable the routine conversion of measurements from DBS samples. Finally, it has been suggested that populations may have become accustomed to home collection of tissue samples due to testing for infection in the pandemic^38^ and we speculate that this may translate to an increased response to a future request for participant led sample collection.

We conclude that while self-collection of DBS samples is feasible, there are lower response rates than in face-to-face sample collection. However, the use of DBS permits large scale collection in an unbiased way that would enable comparable population disease estimates as from venous samples collected by a nurse. We recommend DBS for large scale studies of cardiovascular risk factors collected by participants themselves. Research supports the collection of samples for a wider range of analytes and measures^39^, but this would require additional investigation in population studies.

### Supplementary Information

Supplementary Information.

## Data Availability

Data from the *Understanding Society* Innovation Panel used for this study are available for download from the UK Data Service SN: 6849, http://doi.org/10.5255/UKDA-SN-6849-14.

## References

[1] Pollitt RA, Rose KM, Kaufman JS (2005). Evaluating the evidence for models of life course socioeconomic factors and cardiovascular outcomes: A systematic review. BMC Public Health.

[2] Stringhini S, Zaninotto P, Kumari M, Kivimäki M, Lassale C, Batty GD (2018). Socio-economic trajectories and cardiovascular disease mortality in older people: The English longitudinal study of ageing. Int. J. Epidemiol..

[3] Marmot M, Brunner E (2005). Cohort profile: The whitehall II study. Int. J. Epidemiol..

[4] Elliott P, Peakman TC, Biobank UK (2008). The UK Biobank sample handling and storage protocol for the collection, processing and archiving of human blood and urine. Int. J. Epidemiol..

[5] Fraser A, Macdonald-Wallis C, Tilling K, Boyd A, Golding J, Davey Smith G (2013). Cohort profile: The Avon longitudinal study of parents and children: ALSPAC mothers cohort. Int. J. Epidemiol..

[6] Mills E, Wilson K, Rachlis B, Griffith L, Wu P, Guyatt G (2006). Barriers to participation in HIV drug trials: A systematic review. Lancet. Infect. Dis.

[7] Rockett JC, Buck GM, Lynch CD, Perreault SD (2004). The value of home-based collection of biospecimens in reproductive epidemiology. Environ. Health Perspect..

[8] Williams SR, McDade TW (2009). The use of dried blood spot sampling in the national social life, health, and aging project. J. Gerontol. Ser. B Psychol. Sci. Soc. Sci..

[9] Guyer, H., Ofstedal, M. B., Lessof, C., & Cox, K. The benefits and challenges of collecting physical measures and biomarkers in cross- national studies in March 2022: https://hrs.isr.umich.edu/sites/default/files/biblio/Collecting%20PM-Bio%20Data_DocumentationReport.pdf.

[10] Egier DA, Keys JL, Hall SK, McQueen MJ (2011). Measurement of hemoglobin A1c from filter papers for population-based studies. Clin. Chem..

[11] Affan ET, Praveen D, Chow CK, Neal BC (2014). Comparability of HbA1c and lipids measured with dried blood spot versus venous samples: A systematic review and meta-analysis. BMC Clin. Path..

[12] Crimmins EM, Zhang YS, Kim JK, Frochen S, Kang H, Shim H (2020). Dried blood spots: Effects of less than optimal collection, shipping time, heat, and humidity. Am. J. Hum. Biol..

[13] Hall JM, Fowler CF, Barrett F, Humphry RW, Van Drimmelen M, MacRury SM (2020). HbA1c determination from HemaSpot™ blood collection devices: Comparison of home prepared dried blood spots with standard venous blood analysis. Diabet. Med..

[14] McDade TW, Burhop J, Dohnal J (2004). High-sensitivity enzyme immunoassay for C-reactive protein in dried blood spots. Clin. Chem..

[15] Platt L, Knies G, Luthra RR, Nandi A, Benzeval M (2020). Understanding society at 10 years | European sociological review | Oxford academic. Eur. Sociol. Rev..

[16] Al Baghal, T., *et al.* Collection of biomarkers using nurses, interviewers, and participants: the design of IP12|Understanding Society University of Essex; 2021 in 2021 Oct 14: https://www.understandingsociety.ac.uk/research/publications/546970

[17] McFall SL, Conolly A, Burton J (2014). Collecting biomarkers using trained interviewers. Lessons learned from a pilot study. Surv. Res. Methods.

[18] WhatmanSimpleSpotCheck.pdf in 2021 Oct 14: https://ldh.la.gov/assets/oph/Center-PHCH/Center-PH/genetic/NBSManual/WhatmanSimpleSpotCheck.pdf

[19] mcr.pdf in 2021 Oct 14: https://mran.microsoft.com/snapshot/2014-11-05/web/packages/mcr/mcr.pdf

[20] Deming WE (1943). Statistical Adjustment of Data.

[21] Cornbleet PJ, Gochman N (1979). Incorrect least-squares regression coefficients in method-comparison analysis. Clin. Chem..

[22] Bland JM, Altman DG (1999). Measuring agreement in method comparison studies. Stat. Methods Med. Res..

[23] University of Essex, Institute for Social and Economic Research. Understanding Society: Innovation Panel, Waves 1-12, 2008-2019. [data collection]. 10th Edition. 2021. UK Data Service. SN: 6849. 10.5255/UKDA-SN-6849-13.

[24] Jaszczak A, Lundeen K, Smith S (2009). Using nonmedically trained interviewers to collect biomeasures in a national in-home survey. Field Methods.

[25] Sakshaug JW, Couper MP, Ofstedal MB (2010). Characteristics of physical measurement consent in a population-based survey of older adults. Med. Care.

[26] Korbmacher, J. M. Interviewer effects on respondents’ willingness to provide blood samples in SHARE. SHARE working paper series 20. Available online: WP_Series_20_2014_Korbmacher.pdf (share-project.org).

[27] Kaplan BA (1990). Health and Lifestyles. By Mildred Blaxter Pp. 268 (Tavistock, Routledge, London, 1990) Paperback. J. Biosoc. Sci..

[28] Cernat A, Sakshaug JW, Chandola T, Nazroo J, Shlomo N (2021). Nurse effects on non-response in survey-based biomeasures. Int. J. Soc. Res. Methodol..

[29] Mindell JS, Giampaoli S, Goesswald A, Kamtsiuris P, Mann C, Männistö S (2015). Sample selection, recruitment and participation rates in health examination surveys in Europe—experience from seven national surveys. BMC Med. Res. Methodol..

[30] Pashazadeh, F., Cernat, A., & Sakshaug, J. W. Chapter 16: Investigating the use of nurse paradata in understanding nonresponse to biological data collection. Appendix 16. In: Interviewer effects from a total survey error perspective.

[31] Crimmins E, Kim JK, McCreath H, Faul J, Weir D, Seeman T (2014). Validation of blood-based assays using dried blood spots for use in large population studies. Biodemogr. Soc. Biol..

[32] Lacher, D. A., *et al.* Comparison of dried blood spot to venous methods for hemoglobin A1c, glucose, total cholesterol, high-density - Biospecimen Research Database in Oct 14 2021: https://brd.nci.nih.gov/brd/paper/clin-chim-acta/2013/comparison-of-dried-blood-spot-to-venous-methods-for-hemoglobin/12089 10.1016/j.cca.2013.03.032 23566929

[33] Miller IM, Lacher DA, Chen TC, Zipf GW, Gindi RM, Galinsky AM (2015). Collection and laboratory methods for dried blood spots for hemoglobin A1c and total and high-density lipoprotein cholesterol in population-based surveys. Clin. Chim. Acta.

[34] Fallaize R, Livingstone KM, Celis-Morales C, Macready AL, San-Cristobal R, Navas-Carretero S (2018). Association between diet-quality scores, adiposity, total cholesterol and markers of nutritional status in European adults: Findings from the Food4Me study. Nutrients.

[35] Li W, Zhang J, Tse FLS (2013). Handbook of LC-MS Bioanalysis: Best Practices, Experimental Protocols, and Regulations.

[36] Buxton OM, Malarick K, Wang W, Seeman T (2009). Changes in dried blood spot HbA1c with varied post-collection conditions. Clin. Chem..

[37] Buxton OM, Malarick K, Wang W, Seeman T (2009). Changes in dried blood spot Hb A1c with varied postcollection conditions. Clin. Chem..

[38] Jaklevic MC (2021). Pandemic spotlights in-home colon cancer screening tests. JAMA.

[39] Bhatti P, Kampa D, Alexander BH, McClure C, Ringer D, Doody MM (2009). Blood spots as an alternative to whole blood collection and the effect of a small monetary incentive to increase participation in genetic association studies. BMC Med. Res. Methodol..

