# Supplementary material for: A randomised study of nurse collected venous blood and self-collected dried blood spots for the assessment of cardiovascular risk factors in the Understanding Society Innovation Panel
Source: Sci Rep. 2023 Aug 10;13:13008. doi: 10.1038/s41598-023-39674-6 (PMC10415328; doi:10.1038/s41598-023-39674-6)
Supplement: Supplementary file 1 — Supplementary Information. [file 41598_2023_39674_MOESM1_ESM.docx]

**A randomised study of nurse- and self-collected dried blood spots for the assessment of cardiovascular risk factors in the *Understanding Society* Innovation Panel.**

Meena Kumari*^1^, Alexandria Andrayas^1,2,3^, Tarek Al Baghal^1^, Jonathon Burton^1^, Thomas F. Crossley^4^, Kerry S. Jones^5^, Damon A. Parkington^5^, Albert Koulman^5^, Michaela Benzeval^1^

**Supplementary Materials**

**Supplementary Materials 1: DBS extraction, reagents and assay materials**

**1.1. DBS extraction**

For glycated haemoglobin, one 3.2 mm disc was punched from each DBS directly into the well of a 96-well microtitre plate. Deionised water (50 µL) was added to each well and the plate sealed. Plates were incubated in an ultrasonic bath at room temperature. After 20 minutes, 100 µL of R3 buffer from Randox kit was added and the plate returned to the ultrasonic bath. After 5 minutes, the content of each well was transferred to sample cups for analysis.

For lipid analysis, 2 x 3.2 mm discs were punched into each well of a 96-well plate. 150 µL of methanol was added to each well and the plate covered securely with aluminium foil. The plate was incubated on a plate shaker (700 rpm) for 1 hour at room temperature. Next, 150 µL deionized water was added to each well and mixed by aspiration with the single channel pipette five times before transfer to the sample cup.

For CRP, a 3.2 mm disc was punched from the DBS specimen into a 96-well microtitre plate and eluted in 75 µl phosphate buffered saline (PBS) overnight on a plate shaker set to 600 rpm. The eluents were transferred into sample cups for analysis.

**1.2. Reagents**

Reagents for HbA1C, total cholesterol, (HDL cholesterol) and triglycerides were purchased from Randox Laboratories (County Antrim, UK) and their use adapted for compatibility with Siemens Dimension Xpand clinical chemistry analyser.

**1.2.1. HbA1C**

Reagents: Randox Haemoglobin A1c (HbA1c) (Catalogue number, HA 8043).

Calibrator: Randox Haemoglobin A1c Calibrator, (Catalogue number, HA 3444)

Controls: Streck A1c Cellular (Catalogue number, 211130) as whole blood control, in-house controls 400751, 401261, 400963 (EDTA whole blood spotted onto Whatman 903 DBS card) for DBS elution control.

**1.2.2. Total cholesterol**

Reagents: Randox Cholesterol (Catalogue number, CH 8019).

Calibrator: Randox Calibration Venous Level 3 (Catalogue number, CAL 2351) diluted according to instructions for venous assay and diluted 1:100 with 50:50 methanol/deionised water for DBS assay.

QC: Bio-Rad Laboratories (Watford, UK) Unassayed Multiqual levels 1 & 3 (Catalogue number, 697 & 699) assayed neat for venous assay and diluted 1:100 with 50:50 methanol/deionised water for DBS assay. In-house controls 400330, 401323, 400473 (EDTA Whole blood spotted onto Whatman 903 DBS card) for DBS elution control.

**1.2.3. Triglycerides**

Reagents: Randox triglycerides TR 210.

Calibrator: CAL standard, provided with reagent kit TR 210.

QC: Bio-rad Unassayed Multiqual levels 1 & 3 (Catalogue number, 697 & 699) assayed neat for venous assay and diluted 1:100 with 50:50 methanol/deionised water for DBS assay. In-house controls 400330, 401323, 400473 (EDTA Whole blood spotted onto Whatman 903 DBS card) for DBS elution control.

**1.2.4. C-reactive protein**

Reagents: Siemens CardioPhase® high sensitivity CRP (Catalogue number, RF434)

Calibrator: CardioPhase® high sensitivity CRP (Catalogue number, RC434) used as per instructions for venous assay and diluted 1:50 with PBS for DBS assay.

QC: Bio-Rad Laboratories (Watford, UK) Liquichek elevated CRP control levels 1 (Catalogue number, 254) assayed neat and diluted x2 and x10 in PBS for venous assay and diluted 1:100, 1:200 and 1:500 with PBS for DBS assay. In-house controls D080321, K080321 and M080321 (EDTA whole blood spotted onto Whatman 903 DBS card) for DBS elution control.

**Supplementary Materials 2: Quality control information**

**2.1.1. Lipid analysis quality control**

For quality control (QC), Lyphochek Unassayed Multiqual levels 1 and 3 (Bio-Rad Laboratories (Watford, UK) Catalogue numbers, 697 & 699)) were assayed with each batch to monitor analytical performance. Supplementary Table 2 shows performance of the Multiqual controls with target values taken from the Biorad Unity website (<https://www.qcnet.com/QCNET/UnityReports.aspx>) and derived from worldwide reports from other labs using similar methods. Accuracy and imprecision are acceptable for all serum lipid methods and reflect performance of an assay for use in clinical practice.

*Supplementary Table 2. Lipid liquid QC control results*

|  | **Multiqual 1** | | | **Multiqual 3** | | |
| --- | --- | --- | --- | --- | --- | --- |
|  | **Trig.** | **Chol.** | **HDL** | **Trig.** | **Chol.** | **HDL** |
| **Mean, mmol/L** | 1.04 | 2.89 | 0.68 | 2.28 | 6.67 | 1.57 |
| **SD, mmol/L** | 0.05 | 0.03 | 0.06 | 0.05 | 0.09 | 0.06 |
| **%CV** | 4 | 1 | 8 | 2 | 1 | 4 |
| **N** | 20 | 20 | 25 | 20 | 20 | 24 |
| **Target, mmol/L** | 1.1 | 2.8 | 0.7 | 2.4 | 6.4 | 1.6 |
| **Target Range, mmol/L** | 0.95-1.17 | 2.57-2.93 | 0.48-0.83 | 2.15-2.59 | 5.94-6.80 | 1.21-1.97 |

**2.1.2. Lipid analysis: diluted assay controls**

To better represent expected levels in dried blood spots (DBS) the Multiqual levels 1 and 3 QC materials were assayed with each batch diluted 1:100 in 50% methanol. The dilution used is equivalent to the approximate dilution of serum component of DBS specimens in the elution protocol and the use of these controls allowed the monitoring of assay performance independent of DBS elution at relevant concentrations. As the calibration material used was diluted 1:100 in 50% methanol it was expected that results calculated from analysis of DBS elutions would be equivalent to serum. Assay performance for diluted assay controls are shown in Supplementary Table 3. There was slight inaccuracy for Multiqual 1 (low QC) with average values for triglycerides and cholesterol above the target range for these assays, although these ranges are based on the performance of the serum assay.

*Supplementary Table 3. Lipid diluted liquid QC control results*

|  | **Multiqual 1** | | | **Multiqual 3** | | |
| --- | --- | --- | --- | --- | --- | --- |
|  | **Trig.** | **Chol.** | **HDL** | **Trig.** | **Chol.** | **HDL** |
| **Mean, mmol/L** | 1.25 | 2.98 | 0.57 | 2.51 | 6.74 | 1.68 |
| **SD, mmol/L** | 0.11 | 0.34 | 0.05 | 0.19 | 0.61 | 0.10 |
| **%CV** | 9 | 11 | 8 | 8 | 9 | 6 |
| **N** | 16 | 16 | 16 | 16 | 16 | 16 |
| **Target, mmol/L** | 1.1 | 2.8 | 0.7 | 2.4 | 6.4 | 1.6 |
| **Target Range, mmol/L** | 0.95-1.17 | 2.57-2.93 | 0.48-0.83 | 2.15-2.59 | 5.94-6.80 | 1.21-1.97 |

**2.1.3. Lipids DBS QC**

Three DBS controls were also run with each batch. These were produced in-house by spotting EDTA whole blood onto Whatman 903 filter paper and storing with desiccant at -80°C. DBS QCs were processed alongside the participant samples following the same protocol and allowed the monitoring of assay performance including the elution step.

Supplementary Table 4 shows performance of the three DBS controls. The triglyceride DBS QC shows additional imprecision compared with the Multiqual QC, most likely due to the additional imprecision of the DBS method (including punching of the spot, elution steps etc). Comparison of Multiqual and DBS controls (Supplementary Figure 1) suggests the relative imprecision for HDL-cholesterol and total cholesterol may be caused by sample degradation of the DBS material over time and not analytical imprecision of the method. DBS QCs were run over a time period of 9 months. Batches 1 – 20 were run during January to March 2020, batches 21 – 24 were run during September 2020, the gap between batches was due to a lab closure during covid-19 pandemic.

*Supplementary Table 4. Lipid DBS control results*

|  | **Triglycerides** | | | **Cholesterol** | | | **HDL Cholesterol** | | |
| --- | --- | --- | --- | --- | --- | --- | --- | --- | --- |
|  | **DBS1** | **DBS2** | **DBS3** | **DBS1** | **DBS2** | **DBS3** | **DBS1** | **DBS2** | **DBS3** |
| **Mean, mmol/L** | 2.01 | 1.62 | 3.27 | 4.01 | 6.91 | 5.45 | 0.64 | 0.81 | 0.71 |
| **SD, mmol/L** | 0.24 | 0.27 | 0.50 | 1.16 | 2.03 | 1.62 | 0.27 | 0.33 | 0.29 |
| **%CV** | 12 | 17 | 15 | 29 | 29 | 30 | 42 | 41 | 41 |
| **N** | 22 | 22 | 21 | 22 | 22 | 21 | 21 | 22 | 21 |

**Supplementary** **Figure 1. Plots of liquid (Multiqual) and DBS controls for triglycerides, total cholesterol and HDL cholesterol.**

**
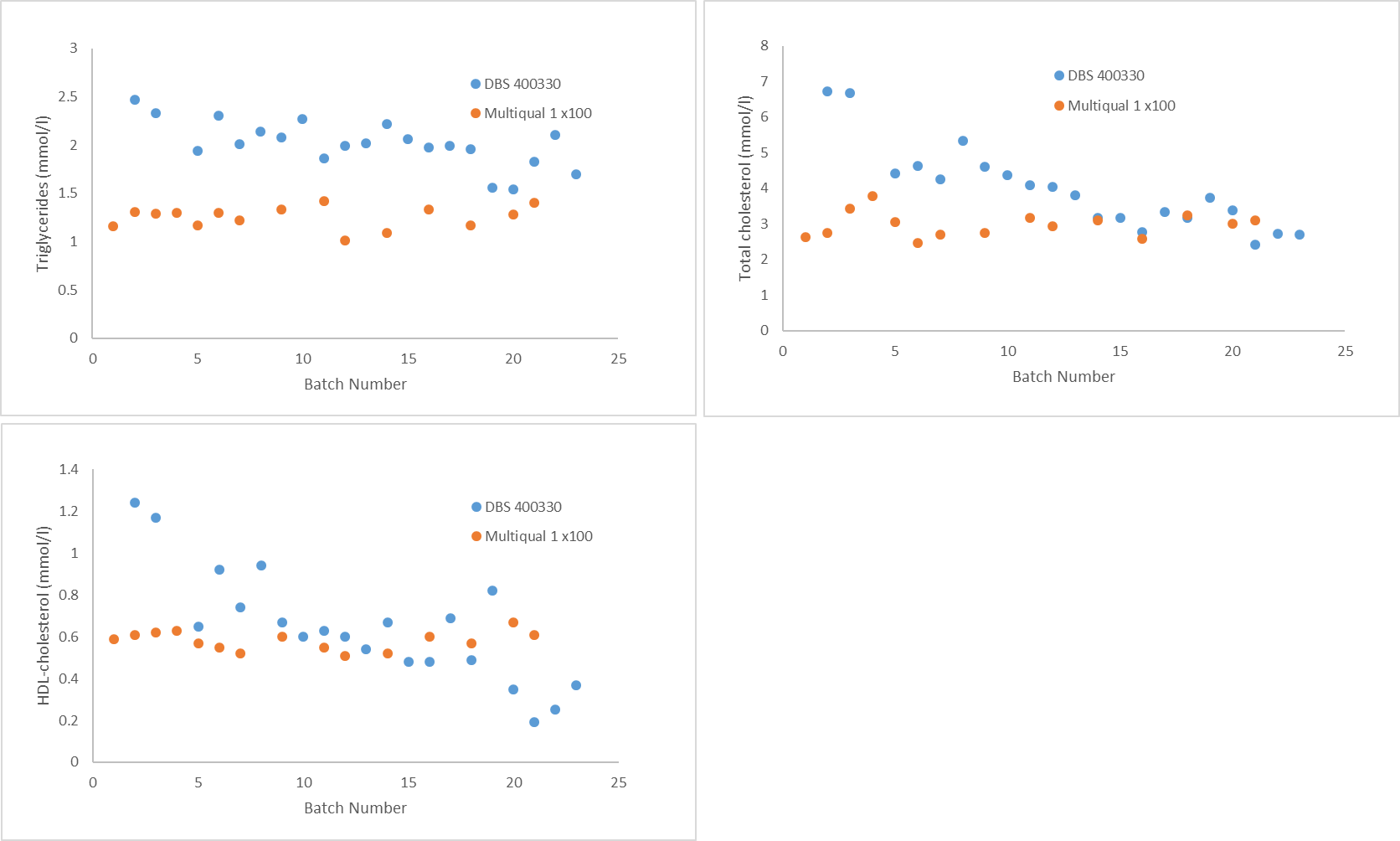
**

**2.2. C-reactive protein (CRP) analysis quality control**

**2.2.1. CRP analysis: serum-equivalent quality control**

For quality control (QC), Liquichek elevated CRP control level 1 (Bio-Rad Laboratories (Watford, UK) Catalogue number 254) was assayed with each batch to monitor analytical performance. The QC material was assayed neat and diluted 1:2 and 1:10 with phosphate-buffered saline. Table 5 shows performance of the Multiqual controls with target values taken from the Biorad Unity website (<https://www.qcnet.com/QCNET/UnityReports.aspx>) and derived from worldwide reports from other labs using similar methods. Accuracy and imprecision are acceptable for all serum lipid methods and reflect performance of an assay for use in clinical practice.

*Supplementary Table 5. CRP liquid QC control results*

|  | **CRP control material** | | |
| --- | --- | --- | --- |
|  | **Neat** | **Diluted 1:2** | **Diluted 1:10** |
| **Mean, mg/L** | 14.3 | 7.6 | 1.8 |
| **SD, mg/L** | 0.6 | 0.3 | 0.0 |
| **%CV** | 5 | 4 | 2 |
| **N** | 13 | 11 | 12 |
| **Target Range, mg/L** | 12.4-19.8 | 6.2-9.9 | 1.2-2.0 |

**2.2.2. CRP analysis: diluted assay controls**

To better represent expected levels in dried blood spots (DBS) the CRP QC material were assayed with each batch diluted diluted st 1:100, 1:200 and 1:500 with PBS for DBS assay. The performance of diluted CRP controls demonstrate acceptable precision of the assay at relevant concentrations (Supplementary Table 6).

*Supplementary Table 6. CRP diluted liquid QC control results*

|  | **CRP control material** | | |
| --- | --- | --- | --- |
|  | Diluted 1:100 | Diluted 1:200 | Diluted 1:500 |
| **Mean, mg/L** | 5.9 | 2.9 | 1.2 |
| **SD, mg/L** | 0.5 | 0.4 | 0.2 |
| **%CV** | 8 | 14 | 16 |
| **N** | 31 | 31 | 31 |
| **Target Range, mg/L** | 4.9 - 6.9 | 2.1 - 3.8 | 0.8 - 1.6 |

**2.2.3. CRP DBS QC**

In-house controls D080321, K080321 and M080321 (EDTA whole blood spotted onto Whatman 903 DBS card) for DBS elution control were also ran with each batch. QCs were processed alongside the participant samples following the same protocol, these controls allowed the monitoring of assay performance including the elution step (Supplementary Table 7). The performance of DBS controls shows greater imprecision particularly at lower concentrations. All DBS CRP batches were run during April to June 2021.

*Supplementary Table 7. CRP DBS QC control results*

|  | DBS CRP controls | | |
| --- | --- | --- | --- |
|  | D080321 | K080321 | M080321 |
| **Mean, mg/L** | 1.5 | 5.5 | 0.8 |
| **SD, mg/L** | 0.4 | 0.7 | 0.3 |
| **%CV** | 25 | 13 | 36 |
| **N** | 24 | 25 | 25 |

**2.3. HbA1c analysis quality control**

**2.3.1. HbA1c analysis: liquid quality control**

Two levels of quality control material (Streck (La Vista, Nebraska, USA) A1c-Cellular control levels 1 and 2 (Catalogue number, 211130)) were assayed during measurement of whole blood and DBS samples. QC materials were diluted 1:41 with haemoglobin denaturant reagent as per the assay protocol and allowed the monitoring of assay performance independent of DBS elution. Target ranges are from product documentation. The same QCs were used for both the whole blood and DBS assays as dilution of DBS samples was equivalent to whole blood assay. Accuracy and imprecision are acceptable and reflect performance of an assay for use in clinical practice (supplementary table 8).

*Supplementary Table 8. HbA1c liquid quality controls*

|  | **STRECK A1C-Cellular controls** | | | |
| --- | --- | --- | --- | --- |
|  | **Measured with whole blood samples** | | **Measured with DBS samples** | |
|  | **Level 1** | **Level 2** | **Level 1** | **Level 2** |
| **Mean (%HbA1c)** | 5.1 | 12.9 | 4.3 | 10.2 |
| **SD (%HbA1c)** | 0.5 | 1.8 | 0.3 | 1.0 |
| **%cv** | 9 | 14 | 7 | 10 |
| **N** | 13 | 9 | 24 | 24 |
| **Target range (%HbA1c)** | 3.8-6.3 | 8.2-13.3 | 3.8-6.3 | 8.2-13.3 |

**2.3.2. HbA1c analysis: in-house whole blood quality control**

Two samples of human EDTA whole blood were included with each batch and diluted 1:41 with haemoglobin denaturant reagent as per the assay protocol (supplementary table 9).

*Supplementary Table 9. HbA1c in-house whole blood quality controls*

|  | Human EDTA WB controls | |
| --- | --- | --- |
|  | 400105 | 401124 |
| **Mean (%HbA1c)** | 4.3 | 10.6 |
| **SD (%HbA1c)** | 0.3 | 0.5 |
| **%cv** | 6 | 4 |
| **N** | 13 | 16 |

**2.3.3. HbA1c analysis: in-house DBS quality control**

Three in-house DBS controls were prepared by spotting EDTA whole blood on to Whatman 903 DBS cards. These QCs were run with each batch, processed alongside the participant samples following the same protocol and allowed the monitoring of assay performance including the elution step. The method performs well in terms of accuracy and precision (Supplementary Table 10). Imprecision is only slightly higher in the DBS samples which suggests HbA1c and Hb are co-eluting proportionally preserving the HbA1c ratio (i.e. the reported value). Batches 1-16 were run during January 2020 to March 2020, batches 17 – 24 were run during September 2020, the gap between batches was due to a lab closure during covid-19 pandemic.

*Supplementary* *Table 10. HbA1c in-house DBS quality controls*

|  | **DBS controls** | | |
| --- | --- | --- | --- |
|  | **400751** | **401261** | **400963** |
| **Mean (%HbA1c)** | 5.3 | 5.5 | 9.4 |
| **SD (%HbA1c)** | 0.3 | 0.3 | 1.0 |
| **%cv** | 5.7 | 5.2 | 10.6 |
| **N** | 24 | 24 | 24 |

**Supplementary Materials 3:**

**Supplementary Figure 2: Distributions of venous-equivalent DBS values for each analyte**

**Supplementary Materials 4:**

**Supplementary Figure 3. Scatter plots showing relationship between venous and venous-equivalent DBS values for each analyte in original units**

**Supplementary Materials 5:**

**Supplementary Figure 4. Bland-Altman plots showing agreement between venous and venous-equivalent DBS values for each analyte in original units**

Supplementary Materials 6: Impact of pre-analytic conditions on analyte measurements

Supplementary Table 11: The effect of DBS and venous characteristics on agreement between venous and venous-equivalent DBS results

| **Covariate** | **Cholesterol (mmol/l), N = 372** | | **C-reactive protein (mg/l), N = 208** | | **Triglycerides (mmol/l), N = 367** | | **HbA1c (%), N = 355** | |
| --- | --- | --- | --- | --- | --- | --- | --- | --- |
|  | **F^*^** | **P** | **F** | **P** | **F** | **P** | **F** | **P** |
| **Number of spots (>= 2 spots vs < 2 spots)** | 1.02 | 0.362 | 3.15 | 0.045 | 2.53 | 0.081 | 13.66 | <0.001 |
| **Quality of spots (Good vs Issue)** | 0.34 | 0.714 | 3.24 | 0.041 | 2.33 | 0.099 | 1.31 | 0.272 |
| **Laboratory batch** | 5.95 | 0.003 | 0.81 | 0.445 | 0.22 | 0.799 | 9.80 | <0.001 |
| **Days at room temperature** | 8.52 | <0.001 | 0.37 | 0.693 | 1.03 | 0.359 | 4.74 | 0.009 |
| **Days between venous blood collection and lab delivery** | 2.36 | 0.095 | 0.17 | 0.847 | 3.00 | 0.051 | 0.18 | 0.835 |
| * Results from ANOVA for full interaction of covariates with DBS on top of the base model of the venous reading regressed against the serum-equivalent DBS result alone | | | | | | | | |

Supplementary Table 12. Beta coefficient and confidence intervals of venous regressed against DBS with an interaction term with number of DBS spots and quality of spots

|  | **Total cholesterol (mmol/l)** | | | **C-reactive protein (mg/l)** | | | **Triglycerides (mmol/l)** | | | **Haemoglobin A1C (%)** | | |
| --- | --- | --- | --- | --- | --- | --- | --- | --- | --- | --- | --- | --- |
| **Characteristic** | **Beta** | **95% CI** | **P** | **Beta** | **95% CI** | **P** | **Beta** | **95% CI** | **p** | **Beta** | **95% CI** | **p** |
| **Number of spots (>= 2 vs < 2)** | | | | | | | | | | | | |
| DBS | 0.49 | 0.48, 0.49 | <0.001 | 1.1 | 1.1, 1.1 | <0.001 | 0.23 | 0.23, 0.23 | <0.001 | 0.51 | 0.50, 0.51 | <0.001 |
| Number of spots |  |  |  |  |  |  |  |  |  |  |  |  |
| => 2 | Ref |  |  | Ref |  |  | Ref |  |  | ref |  |  |
| < 2 | -0.55 | -0.58, -0.52 | <0.001 | 0.46 | 0.43, 0.49 | <0.001 | -0.42 | -0.43, -0.41 | <0.001 | 1.6 | 1.6, 1.7 | <0.001 |
| DBS * Number of spots |  |  |  |  |  |  |  |  |  |  |  |  |
| DBS * < 2 | 0.07 | 0.07, 0.08 | <0.001 | 0.03 | 0.02, 0.04 | <0.001 | 0.11 | 0.10, 0.11 | <0.001 | -0.36 | -0.36, -0.35 | <0.001 |
| **Quality of DBS spots (Good vs Issue)** | | | | | | | | | | | | |
| DBS | 0.53 | 0.53, 0.53 | <0.001 | 1.1 | 1.1, 1.1 | <0.001 | 0.27 | 0.27, 0.27 | <0.001 | 0.44 | 0.44, 0.44 | <0.001 |
| DBS quality |  |  |  |  |  |  |  |  |  |  |  |  |
| Good | Ref |  |  | Ref |  |  | Ref |  |  | ref |  |  |
| Issue | 0.46 | 0.44, 0.49 | <0.001 | 0.17 | 0.15, 0.19 | <0.001 | 0.02 | 0.01, 0.03 | <0.001 | 0.68 | 0.65, 0.70 | <0.001 |
| DBS * DBS quality |  |  |  |  |  |  |  |  |  |  |  |  |
| DBS * Issue | -0.09 | -0.09, -0.08 | <0.001 | -0.17 | -0.18, -0.17 | <0.001 | -0.10 | -0.10, -0.10 | <0.001 | -0.14 | -0.14, -0.13 | <0.001 |

Supplementary Table 13. Beta coefficient and confidence intervals of venous regressed against DBS with an interaction term with days DBS stored at room temperature, DBS sample delays between venous lab delivery, and laboratory batch (pre/post COVID closure)

**Supplementary Figure SEQ Supplementary_Figure \* ARABIC 3: Correlation matrices**

|  | **Total cholesterol (mmol/l)** | | | **C-reactive protein (mg/l)** | | | **Triglycerides (mmol/l)** | | | **Haemoglobin A1C (%)** | | |
| --- | --- | --- | --- | --- | --- | --- | --- | --- | --- | --- | --- | --- |
| **Characteristic** | **Beta** | **95% CI** | **P** | **Beta** | **95% CI** | **P** | **Beta** | **95% CI** | **p** | **Beta** | **95% CI** | **p** |
| **Laboratory batch** | | | | | | | | | | | | |
| DBS | 0.61 | 0.60, 0.61 | <0.001 | 1.1 | 1.1, 1.1 | <0.001 | 0.26 | 0.25, 0.26 | <0.001 | 0.50 | 0.50, 0.50 | <0.001 |
| Laboratory batch |  |  |  |  |  |  |  |  |  |  |  |  |
| Before closure | — | — |  | — | — |  | — | — |  | — | — |  |
| After closure | 1.5 | 1.4, 1.5 | <0.001 | -0.87 | -0.89, -0.85 | <0.001 | 0.14 | 0.14, 0.15 | <0.001 | 0.12 | 0.09, 0.15 | <0.001 |
| DBS * Laboratory batch |  |  |  |  |  |  |  |  |  |  |  |  |
| DBS * After closure | -0.23 | -0.24, -0.23 | <0.001 | 0.19 | 0.19, 0.20 | <0.001 | -0.06 | -0.06, -0.06 | <0.001 | -0.08 | -0.08, -0.07 | <0.001 |
| **Days stored at room temperature** | | | | | | | | | | | | |
| DBS | 0.43 | 0.42, 0.44 | <0.001 | 1.3 | 1.3, 1.3 | <0.001 | 0.23 | 0.22, 0.23 | <0.001 | 0.13 | 0.12, 0.14 | <0.001 |
| Days stored at room temp. | -0.02 | -0.02, -0.01 | <0.001 | 0.01 | 0.01, 0.01 | <0.001 | -0.003 | -0.003, -0.003 | <0.001 | -0.02 | -0.02, -0.02 | <0.001 |
| DBS * Days stored at room temp. | 0.001 | 0.001, 0.001 | <0.001 | -0.002 | -0.002, -0.002 | <0.001 | 0.0002 | 0.0001, 0.0003 | <0.001 | 0.004 | 0.004, 0.004 | <0.001 |
| **Days between venous collection and lab delivery** | | | | | | | | | | | | |
| DBS | 0.69 | 0.69, 0.70 | <0.001 | 1.1 | 1.1, 1.1 | <0.001 | 0.11 | 0.10, 0.11 | <0.001 | 0.46 | 0.45, 0.46 | <0.001 |
| Days between venous collection and lab delivery | 0.30 | 0.30, 0.31 | <0.001 | 0.05 | 0.05, 0.06 | <0.001 | -0.04 | -0.04, -0.04 | <0.001 | 0.08 | 0.07, 0.09 | <0.001 |
| DBS * Days between venous collection and lab delivery | -0.05 | -0.05, -0.05 | <0.001 | 0.00 | 0.00, 0.00 | <0.001 | 0.04 | 0.04, 0.04 | <0.001 | -0.02 | -0.02, -0.01 | <0.001 |

**Supplementary material 7:**

Supplementary table 14: Outcomes for participants contacted and reasons for non-participation.

| **Outcomes following contact** | | N= |
| --- | --- | --- |
| full interview | | 2162 |
| proxy interview | | 51 |
| CAPI interview lost | | 3 |
| Refusal |  | 312 |
| other non-interview | | 219 |
| Illness or away during survey period | | 8 |
| Elderly or too infirm to participate | | 9 |
| Refusal (non-interviewing household) | | 890 |
| Elderly or too infirm (non-interviewing household) | | 10 |
| Illness or away (non-interviewing household) | | 46 |
|  |  |  |
|  |  | 3710 |

**Supplementary Materials 8:**

**Supplementary Figure 5. Scatter plot showing venous against raw DBS data for HDL-cholesterol**

Supplementary Materials 9:

Supplementary Table 15. Proportion of doctor diagnosed diabetes by mode of sample collection in different subgroups

|  |  |  |  |
| --- | --- | --- | --- |
| **Characteristic** | **Total N** | **Nurse** | **Self-collection** |
| **Full interview** | 2,049 | 62 (9.8%) | 122 (8.6%) |
| **Consent to DBS** | 984 | 47 (9.8%) | 55 (10.8%) |
| **DBS analysed** | 909 | 43 (9.5%) | 44 (9.7%) |
| **HbA1c DBS analysed** | 885 | 38 (8.5%) | 38 (8.7%) |
| **HbA1c DBS not analysed (excluding outliers)** | 24 | 5 (55.5%) | 6 (40.0%) |
| **HbA1c DBS not analysed (outliers)** | 10 | 0 (0.0%) | 0 (0.0%) |
| **Consent to venous blood** | 445 | 43 (9.7%) | - |
| **Venous blood analysed** | 394 | 34 (8.6%) | - |
| **HbA1c venous analysed** | 368 | 21 (5.7%) | - |
| **HbA1c venous not analysed (excluding outliers)** | 26 | 13 (50%) | - |
| **HbA1c venous not analysed (excluding outliers)** | 15 | 3 (20%) | - |
